# The density and spatial arrangement of the invasive oyster *Crassostrea gigas* determines its impact on settlement of native oyster larvae

**DOI:** 10.1002/ece3.872

**Published:** 2013-12-10

**Authors:** Emma M Wilkie, Melanie J Bishop, Wayne A O'Connor

**Affiliations:** 1 Department of Biological Sciences, Macquarie UniversityNorth Ryde, NSW, 2109, Australia; 2 NSW Department of Primary Industries, Port Stephens Fisheries InstituteTaylors Beach, NSW, 2316, Australia

**Keywords:** *Crassostrea gigas*, density, filtration, spatial arrangement, surface area.

## Abstract

Understanding how the density and spatial arrangement of invaders is critical to developing management strategies of pest species. The Pacific oyster, *Crassostrea gigas*, has been translocated around the world for aquaculture and in many instances has established wild populations. Relative to other species of bivalve, it displays rapid suspension feeding, which may cause mortality of pelagic invertebrate larvae. We compared the effect on settlement of Sydney rock oyster, *Saccostrea glomerata*, larvae of manipulating the spatial arrangement and density of native *S. glomerata*, and non-native *C. gigas*. We hypothesized that while manipulations of dead oysters would reveal the same positive relationship between attachment surface area and *S. glomerata* settlement between the two species, manipulations of live oysters would reveal differing density-dependent effects between the native and non-native oyster. In the field, whether oysters were live or dead, more larvae settled on *C. gigas* than *S. glomerata* when substrate was arranged in monospecific clumps. When, however, the two species were interspersed, there were no differences in larval settlement between them. By contrast, in aquaria simulating a higher effective oyster density, more larvae settled on live *S. glomerata* than *C*. *gigas*. When *C. gigas* was prevented from suspension feeding, settlement of larvae on *C. gigas* was enhanced. By contrast, settlement was similar between the two species when dead. While the presently low densities of the invasive oyster *C. gigas* may enhance *S. glomerata* larval settlement in east Australian estuaries, future increases in densities could produce negative impacts on native oyster settlement. *Synthesis and applications*: Our study has shown that both the spatial arrangement and density of invaders can influence their impact. Hence, management strategies aimed at preventing invasive populations reaching damaging sizes should not only consider the threshold density at which impacts exceed some acceptable limit, but also how patch formation modifies this.

## Introduction

Biological invasions represent one of the greatest threats to coastal marine ecosystems and their important economic values (Grosholz 2002). They can reduce marine biodiversity, cause fisheries collapses, foul infrastructure, and produce changes to ecosystem processes, such as nutrient cycling and productivity (Vitousek et al. 1996; Bax et al. 2003; Rilov and Crooks 2009). Already, marine invaders number over 320 globally (Molnar et al. 2008). Despite increasingly rigorous quarantine procedures, transport via international shipping and deliberate introduction for fisheries continue to contribute to new invasions every year (Bax et al. 2003).

The establishment of non-native species poses a considerable problem to managers because, once there, they are often impossible to eradicate (Vitousek et al. 1996). Consequently, management of invasive species often necessarily revolves around keeping invader populations at sufficiently low abundance that the economic benefits of the population control exceed the financial costs (Finnoff et al. 2005; Whittle et al. 2007). Yet although impacts of invaders generally increase with abundance (Escapa et al. 2004; Griffen and Byers 2009; Padilla 2010), this relationship is not necessarily linear (see Yokomizo et al. 2009). This is because impact may be evident even at low abundance where the spatial arrangement of an invasive species within a landscape produces locally high density (Bell et al. 1995; Flather and Bevers 2002; Matias et al. 2010, 2011). Disentangling the impact of the spatial arrangement of an invader versus abundance is, therefore, critical if limited management resources are to be most effectively put toward invader control.

Invasive habitat-forming bivalves are among the taxa that can have disproportionately large ecological impacts relative to their abundance. In environments where hard surfaces are limited, bivalve shells increase attachment substrate, and interstitial spaces that protect organisms from predators, waves, sedimentation, and desiccation (Gutiérrez et al. 2003; Commito et al. 2008). Consequently, where habitat-forming invaders increase the habitat complexity of an environment, they are predicted to increase the abundance and diversity of associated organisms (Crooks 2002) – a function that would, presumably, increase with their abundance.

As suspension feeders, bivalves, however, also have the potential to impact communities by influencing patterns of larval settlement (Tamburri et al. 2007; Troost et al. 2009). The majority (∼70%) of coastal sessile species have a planktonic stage in their life history (Jones et al. 1999), and invasive bivalves may transform the distributions and abundance of such organisms by altering dispersal trajectories and survival through to settlement (Gribben et al. 2009; Woodford and McIntosh 2010). Whereas solitary bivalves may enhance settlement by producing feeding currents that entrain larvae (Tamburri et al. 2007), the stronger currents of clusters of bivalves might lead to ingestion and, in some cases, consumption of larvae (Smaal et al. 2005; Troost 2010). Where negative effects of currents produced during suspension feeding overwhelm positive effects of bivalve habitat complexity, and innate recognitions of parent chemical cues by their larvae (e.g., Zimmer and Butman 2000; Elbourne et al. 2008), reductions in the abundance and diversity of native flora and fauna may result.

The Pacific oyster *Crassostrea gigas* (Ostreidae) is the most translocated marine taxa in the world for aquaculture due to its rapid growth, maturation, and broad environmental tolerances (Nell and Perkins 2005; Troost 2010). It has been introduced to over 66 non-native regions for aquaculture and become a nuisance invasive species in 24 regions (Ruesink et al. 2005), leading to major shifts in associated community structure (Markert et al. 2009; Troost et al. 2009). In Australia, *C. gigas* was initially introduced to the southern states for aquaculture in the 1940s and 1950s (Medcof and Wolf 1975). In 1984–1985, wild *C. gigas* appeared in Port Stephens, New South Wales, after a suspected rogue introduction, growing to a population of 26 million by 1988 (Nell 1993). Subsequently, the Pacific oyster has established breeding populations in most NSW estuaries south of the Macleay River (Pacific Oyster Survey 2010). To reduce the potential for further proliferation, culture of *C. gigas* is predominant of sterile triploid individuals (O'Connor and Dove 2009).

In southeastern Australia, wild *C. gigas* typically live alongside the native Sydney rock oyster, *Saccostrea glomerata* (Summerhayes et al. 2009; Bishop et al. 2010). Yet although in most estuaries the total abundance of the non-native is low, it can form monospecific clumps, accounting for up to 100% of oysters at the patch scale (Bishop et al. 2010; Wilkie et al. 2013). *Saccostrea glomerata* and *C. gigas* each form habitat of similar complexity (Wilkie et al. 2012), but *C. gigas* is a more rapid filterer (Bayne 2002). The two species may also produce different chemical cues that might influence settlement processes. Hence, the two species may not be equivalent in their net effect on settlement processes of marine invertebrates.

Here, we investigate: (1) whether the two oyster species, *S. glomerata* and *C. gigas*, provide equal settlement opportunities for *S. glomerata* larvae, when present in mixtures and in monocultures and when at higher and lower density; and (2) whether any differences in *S. glomerata* settlement between the two species are caused by differences in suspension feeding process between the two oysters. We predict that at low densities, or in mixed oyster beds, the strong feeding currents produced by live *C. gigas* may enhance larval settlement over that on low density *S. glomerata*. Where live *C. gigas* are present at high density, or as a monoculture, we expect, however, that the net effect of *C. gigas* suspension feeding will be larval ingestion or entrapment in feeding currents resulting in decreased larval settlement on the non-native as compared to *S. glomerata*. We expect that settlement on dead oyster shells will be proportional to substrate area for attachment and that this relationship will be similar between *C. gigas* and *S. glomerata*. Understanding the functional equivalency of the two species is critical in developing management strategies to minimize impacts of non-native *C. gigas* on native biodiversity.

## Methods

### Larval cultures and adult oysters

The *S. glomerata* oyster larvae used in experiments were produced at the Port Stephens Fisheries Institute (Taylors Beach, NSW, Australia). Larvae were large enough to be retained on a 212 *μ*m screen, could swim, had developed eye spots, 3–5 gill buds and a protruding foot which indicates that they are competent to settle, and were between 20 and 22 days old (O'Connor and Dove 2009).

**Figure 1:**
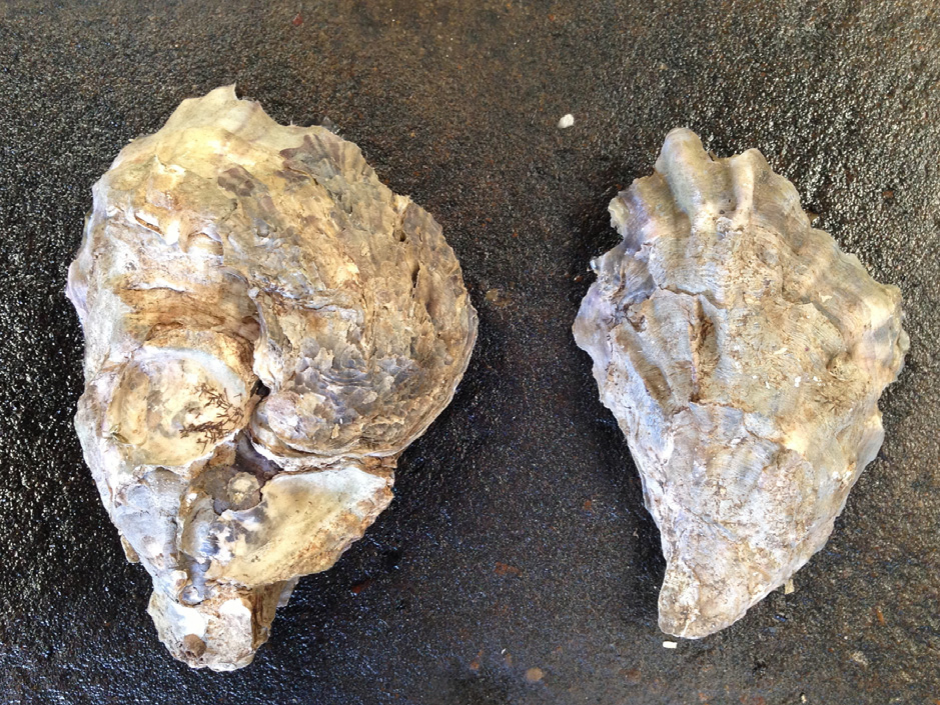
Non-native Pacific oyster *Crassostrea gigas* (left) heavily fouled by juvenile oyster “spat,” and native Sydney rock oyster *Saccostrea glomerata* (right), moderately fouled.

Adult *S. glomerata* and *C. gigas* used as settlement substrates were sourced from aquaculture leases in Port Stephens (Fig. 1). Unless otherwise indicated, all oysters were cleaned of fouling organisms by scrubbing with a wire brush prior to experiments. For each type of oyster, the average shell height (distance between hinge and ventral margin) was calculated from 30 individuals, and the average shell surface area was calculated from 10 individuals, per experiment. To calculate shell surface area, we wrapped replicate oysters in aluminum foil, covering all exposed surface, and measured the surface area (mm^2^) of the resulting piece of foil using ImageJ software (National Health Institute). Although, within each experiment, the shell height of the various types of oyster shell-substrate were matched, we standardized larval settlement by mean shell surface area of each treatment to remove any confounding effect of slight differences.

### Equivalence of native and non-native oysters as substrates for *Saccostrea glomerata* settlement in the field

We conducted experimental field deployments of live and dead oysters to address (1) whether natural settlement of oyster larvae differs between *C. gigas* and *S. glomerata* and (2) how differences in settlement between substrates are dependent on spatial arrangement of oysters. Deployments were within Salamander Bay, Port Stephens, NSW (32°43′S, 152°07E), at a site at which oyster farmers collect *S. glomerata* recruits for farming due to the good larval supply. We hypothesized that differences in larval settlement would be more pronounced between *C. gigas* and *S. glomerata* when the oysters were live and actively feeding than dead, and when they were presented in monocultures, rather than mixed-species arrangements that may dilute feeding-current pressures, chemical cues, and other substrate-specific effects.

Differences in settlement between monospecific clumps of *S. glomerata* and *C. gigas* were tested by deploying nine oysters of a single species [shell height (mean ± SE): *S. glomerata* 79 ± 1.0 mm; *C. gigas* 81 ± 1.5 mm] in polyethylene mesh bags. For each oyster species, we established replicate bags comprised only of live oysters and replicate bags comprised only of dead oysters. For each of the four resulting treatments, there were four replicate bags (i.e., 16 bags in total). Bags containing oysters were suspended at 0.5 m above low-water springs. To assess whether any differences in settlement of oysters between substrates were still apparent when the oyster substrates were interspersed, such as in a mixed-species bed, we deployed 20 oysters of each species and of each status (live or dead; i.e., 80 oysters total) in a single (850 × 400 × 220 mm) SEAPA long-line oyster basket, at a tidal elevation of 0.5 m above low-water springs.

For each of the deployments, dead oysters were produced by carefully opening live oysters, so as to avoid shell damage, removing all tissue and gluing shut the two valves with nontoxic, insoluble two-part epoxy resin adhesive (Araldite, Thornleigh, NSW, Australia). A similar quantity of glue was applied to each live oyster, without sealing the valves shut, to control for any effect of the resin on oyster settlement. Consequently, dead oysters were structural mimics of live oysters, but without the capacity to siphon water. Oyster substrates were deployed in February (during which time historical records indicate recruitment is highest) of 2011 and retrieved after 4 months. The number of recruits on the external surface of each oyster was enumerated. At the time of retrieval, recruits were still too small to readily distinguish between *S. glomerata* and *C. gigas* on the basis of external morphology. Although from historical patterns of larval settlement at this site, *S. glomerata* likely dominated new recruits (G. Diemar, pers. comm.).

To compare larval settlement among monoculture bags, we used a 3-way analysis of variance (ANOVA) with the factors: species (two levels: *S. glomerata*, *C. gigas*); status (two levels: live, dead) and bags (nested within species and status; four levels, random), with *n* = 9 oysters per bag. Settlement was compared among treatments contributing to the mixed-species basket using a 2-way ANOVA with orthogonal factors: species (two levels: *S. glomerata*, *C. gigas*) and status (two levels: live, dead), with *n* = 20 oysters. Cochran's *C*-tests were run prior to each test to assess homogeneity of variance. Where data were not homogeneous, we performed √(*x* + 1) transformations, which are recommended for Poisson-distributed counts data (Sokal and Rohlf 1995; Underwood 1997; Maindonald and Braun 2007), following which assumptions of homogeneity of variance were met. Post hoc Tukey's tests examined the sources of significant treatment effects at *α* = 0.05.

### Equivalence of native and non-native oysters as substrates for *Saccostrea glomerata* settlement in aquaria

To test whether at higher effective densities than those presently attained by *C. gigas* in the field, (1) live *C. gigas* and *S. glomerata* would differ in their density-dependent effects on *S. glomerata* settlement; but (2) dead oysters of each two species would similarly support an increasing settlement of oysters with increasing abundance, we conducted a laboratory experiment. The experiment had three factors, oyster status (two levels: live, dead), species (two levels: *S. glomerata*, *C. gigas*), and density [three levels: high (H), three oysters; medium (M), two oysters; and low (L), one oyster per 8-L aquaria, *n* = 5]. We predicted that for live oysters, the relationship between density and larval settlement would be nonlinear because at low oyster densities, filtration would facilitate larval settlement by drawing larvae in, but at high oyster densities, filtration would inhibit larval settlement through larval ingestion. We expected the switch from positive to negative effects to be at a lower density threshold for *C. gigas*, which has a greater filtration rate. By contrast, we expected that the relationship between the density of dead oysters and settlement would be linear and directly related to surface area.

Dead oyster shells were produced by shucking live oysters and removing all tissue. The shucking method was used to maintain the biofilm, which may influence larval settlement (Tamburri et al. 1992). Hence, the main source of difference between live and dead oysters was the ability to filter. Oysters were positioned in the center of an 8-L aquaria with 750 mL aerated filtered seawater, which was changed after 2 days. We pipetted 1000 *S. glomerata* eyed larvae (0.125/mL) into the center of each aquaria, at the water line. The larvae in each aquarium were retained on a 212-*μ*m sieve then washed back into the same aquarium to continue the experiment. The experiment lasted 4 days, which is sufficient time for larvae to settle (O'Connor and Dove 2009). At the conclusion of the experiment, each shell was examined under a stereomicroscope, and the settled larvae were counted.

To test for interacting effects of the species, and the density of the substrate on larval settlement, we performed fully orthogonal ANOVAs with the two factors, species (two levels: *S. glomerata* and *C. gigas*) and density (three levels: H, M, and L). Results from the experiments using live and dead oysters were analyzed separately because larvae were able to settle on the inside surface of dead oyster shells, and the substrate available for settlement hence differed between the two experiments. Analyses testing for differences in larval settlement among live oyster treatments were run on ranked abundances of larvae to eliminate issues of heterogeneous variances resulting from highly variable settlement. Raw counts were analyzed to test hypotheses about settlement on dead oyster shells.

### Effects of oyster filtration on larval settlement

To test whether differences in filtration rate between live *C. gigas* and *S. glomerata* contribute to differences in numbers of *S. glomerata* recruiting to their surfaces, we (1) ascertained differences in filtration rate between the two species; and (2) assessed whether the difference in the number of larvae settling on live *S. glomerata* and *C. gigas* adults at high density was less when feeding was inhibited than when it was allowed.

Live oysters were restricted from filtering by tightly binding individuals shut with a rubber band (herein “banded” oysters). To determine differences in oyster filtration rates between *S. glomerata* and *C. gigas* (L/h), and the efficacy of the rubber banding in preventing filtration, we used an algal depletion experiment (Coughlan 1969). Twenty similar-sized oysters of each species (shell height; SR: 87 ± 2 mm; PO: 96 ± 2 mm) were placed in filtered seawater, and the first ten of each to gape and commence feeding were selected for use in the experiment. Individuals were randomly assigned to one of two treatments: untouched and banded (*n* = 5 oysters of each species per treatment). Each individual was placed in a separate 8-L aquarium with aerated filtered seawater. Oysters were fed a concentration of 100,000 Tahitian *Isochrysis aff. galbana* algae cells per mL. Every 30 min, a 1-mL aliquot of water from each individual aquarium was collected to determine the algal concentration, and hence the number of algal cells consumed. Following each sample collection, consumed algae were replenished to maintain a constant concentration of 100,000 Tahitian *Isochrysis* algae cells per mL throughout the experiment. After 3 h, the experiment was terminated and the total number of algal cells consumed was calculated.

To assess species-specific effects of filtration on *S. glomerata* settlement, we conducted a fully orthogonal experiment, with the factors; species (two levels: *S. glomerata* and *C. gigas*) and treatment (three levels: nonbanded, banded, and banding control). We hypothesized that a greater number of larvae would settle on the banded than the nonbanded oysters, and the difference between banded and nonbanded treatments would be greater for *C. gigas* than *S. glomerata*. The banding control treatment, where oysters were loosely bound by rubber bands that did not restrict filtering, allowed us to ascertain whether the structure or composition of the rubber influenced larval settlement. Three oysters of a single species were used per replicate. There were five replicates of each treatment. Methods for the experiments were otherwise as described for the density experiment.

A two-way fully orthogonal ANOVA with the factors banding (two levels: banded vs. untouched) and species (two levels: *S. glomerata* vs. *C. gigas*) tested whether there were differences in filtration rate (L/h) between the two species that were reduced by banding the oysters. Analogous ANOVAs, but also including a third level of the factor banding (banding control), tested for effects of oyster type and treatment on total larval settlement.

## Results

### Equivalence of native and non-native oysters as substrates for *Saccostrea glomerata* settlement in the field

**Figure 2:**
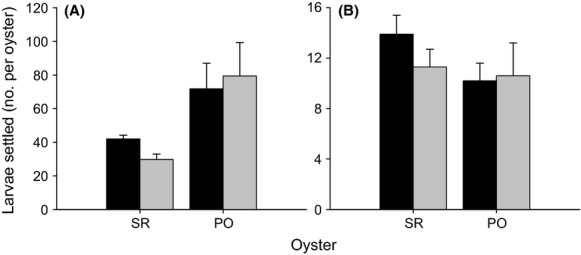
Mean (±SE) *Saccostrea glomerata* recruits per live (black) or dead (gray) oyster in (A) monospecific bags each containing nine Sydney rock (SR) or Pacific (PO) oysters (*n* = 4 bags) and (B) a mixed-species basket containing each species, with *n* = 20 individual oysters per treatment, thus a total of 80 oysters.

In the field, the spatial arrangement of oyster substrates influenced patterns of settlement on *C. gigas* and *S. glomerata* (Fig. 2A,B). Despite significant variation in larval settlement among bags, effects of oyster species and status were evident (Table 1). More larvae settled in monospecific bags of *C. gigas* than in monospecific bags of *S. glomerata* irrespective of status as live or dead (Table 1, Fig. 2A), and for each species, settlement was higher on live than dead oysters (Table 1, Fig. 2A). When, however, oyster treatments were interspersed in a basket, differences in settlement were not apparent between *C. gigas* or *S. glomerata* between live and dead substrates, or an interaction of the two (Table 1, Fig. 2B).

**Table 1:** Analyses of variance testing for differences in the number of *Saccostrea glomerata* larvae settled on wild stock *S. glomerata* (SR), and *Crassostrea gigas* (PO) oyster substrates in field deployed monocultures (*n* = 9 oysters) and mixed-species trays (*n* = 20 oysters).

Source	Monospecific				Mixed			
	
	df	MS	*F*	*P*	df	MS	*F*	*P*
Oy	1	126.09	16.00	**<0.01**	1	0.18	4.81	0.27
S	1	43.41	5.51	**<0.05**	1	0.60	17.06	0.15
B (Oy, S)	12	7.88	3.20	**<0.01**	na	na	na	na
Oy × S	1	22.60	2.87	0.11	1	0.04	0.04	0.85
Res	128	2.46			76	1.00		

Tukey's tests	
Oy	*C. gigas* > *S. glomerata*
S	Live > dead

Oy, oyster (two levels: *S. glomerata and C. gigas*); S, status (two levels, live or dead); B, bag (four levels, random). Data were √(*x* + 1) transformed prior to analysis. Significant differences at are highlighted in bold.

### Effect of substrate density on larval settlement and survival

**Figure 3:**
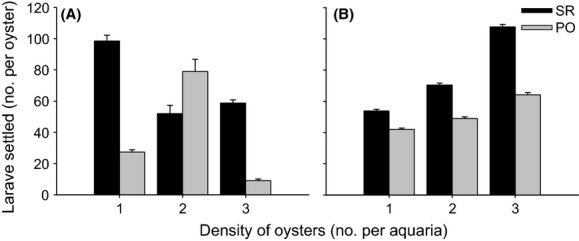
Mean (±SE) *Saccostrea glomerata* larvae settling per oyster in 8-L aquaria with 1, 2, or 3 (A) live or (B) dead Sydney rock (SR) or Pacific (PO) oysters. *n* = 5.

In aquaria, as in the field, the influence of density and type of substrate on larval settlement was dependent on whether the substrate was live or dead. In contrast to the field, however, settlement of *S. glomerata* on live oyster substrate in the 8-L aquarium experiment was greater on *S. glomerata* than *C. gigas* (ANOVA: *F*_1,24_ = 7.61, *P* < 0.05; Fig. 3A). This pattern was driven by the difference in settlement on *S. glomerata* and *C. gigas* at the low density of live oysters (Tukey's test, *P* < 0.05). There was no significant difference in larval settlement between the two species at the medium or the high density of live oysters (*P* > 0.05). There was no effect of species on settlement when the oysters were dead (ANOVA: *F*_1,24_ = 0.86, *P* > 0.05, Fig. 3B). Among dead oyster treatments, significantly more larvae settled in the high-density treatments than in the medium- or low-density treatments, irrespective of species (ANOVA: *F*_2,24_ = 5.23, *P* < 0.01, Fig. 3B).

### Effects of oyster filtration on larval settlement

Rates of water filtration differed between oyster species (ANOVA: *F* = 53.89, *P* < 0.001). *C. gigas* filtered water approximately twice as fast *S. glomerata* (mean ± SE; *C. gigas*: 1.09 ± 0.08 L/h; *S. glomerata*: 0.46 ± 0.05 L/h). The banding shut of oysters produced species-specific reductions in their filtration rate (ANOVA, sig. banding x species interaction: *F*_2,24_ = 5.01, *P* < 0.05). Banded *C. gigas* filtered water significantly slower than nonbanded *C. gigas* (banded: 0.00 ± 0.28 L/h; nonbanded: 1.09 ± 0.08 L/h, Tukey's tests, *P* < 0.05), but the filtration rate of *S. glomerata*, which was much lower than that of *C. gigas*, did not significantly differ between banded and nonbanded oysters, despite banding halving the mean filtration rate (banded: 0.23 ± 0.06 L/h; nonbanded: 0.46 ± 0.05 L/h; Tukey's tests: *P* > 0.05).

**Figure 4:**
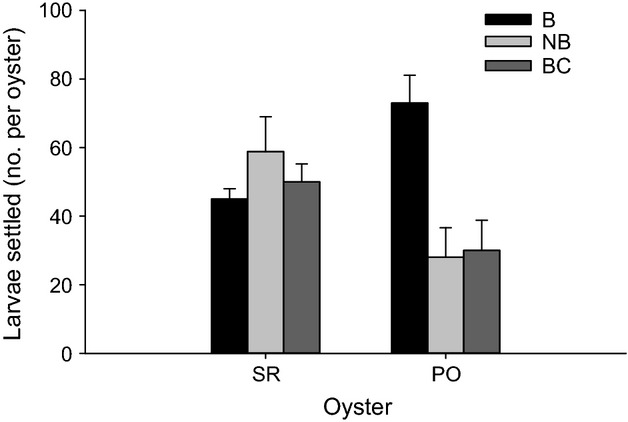
Mean (±SE) *Saccostrea glomerata* larvae that settled per oyster on clusters of either three Sydney rock (SR) or Pacific (PO) oysters that were banded and unable to filter (B), nonbanded and able to filter (NB), or banded controls that were able to filter (BC). Larvae settled were counted on the whole oyster (A) and the proportion (%) of larvae settled within 1 mm of the oyster gaping valve (B). *n* = 5.

Settlement of *S. glomerata* was determined by the interacting effect of oyster species and the banding treatment (ANOVA: *F*_2,24_ = 5.01, *P* < 0.05, Fig. 4). For both species, there was no difference in settlement between the nonbanded and the loosely banded control oysters (Tukey's tests, *P* > 0.05), such that the effects of banding could be interpreted as an effect of reducing filtration, and not of other confounding factors. A significantly greater number of larvae settled on banded than on control or nonbanded *C. gigas* (Tukey's tests, *P* < 0.05). There was no significant difference in the number of larvae that settled on banded, control, or nonbanded *S. glomerata* (Tukey's tests, *P* > 0.05). Between species, significantly more larvae settled on nonbanded *S. glomerata* than *C. gigas* (Tukey's tests, *P* < 0.01), while among banded oysters, there were no differences in larval settlement between species (Tukey's tests, *P* > 0.05).

## Discussion

Through a combination of laboratory and field experiments, we have demonstrated that the impact of non-native *Crassostrea gigas* on settlement of native *Saccostrea glomerata* larvae is dependent on the non-native oyster's spatial arrangement and density. In the field, ∼ 100% more larvae settled on *C. gigas* than *S. glomerata*, when the two oyster species were presented as clusters of a single species. When, however, *C. gigas* was interspersed with *S. glomerata*, settlement of *S. glomerata* did not differ between the two oyster substrates and was similar to settlement on single-species clusters of *S. glomerata*, of equivalent density. At the higher effective density and low-flow environment within aquaria, the impact of *C. gigas* reversed, with 600% less settlement of *S. glomerata* larvae on *C. gigas* relative to *S. glomerata*.

The reason why, among still-water aquaria containing only a single oyster substrate, fewer *S. glomerata* larvae settled in aquaria with *C. gigas* than *S. glomerata* appeared to be the greater filtration rate of the non-native than the native oyster. In the absence of external currents, localized currents produced by suspension feeders can be the primary determinant of settlement patterns (Abelson and Denny 1997). In concordance with a previous study (Bayne 2002), we found that the filtration rate of *C. gigas* was roughly twice that of *S. glomerata*. When monocultures of oysters were free to filter, greater numbers of larvae settled on clusters of *S. glomerata* than *C. gigas*. When, however, filtration was prevented, either by killing the oysters, or banding shut their valves, this pattern disappeared and, in some cases, was reversed with greater settlement on dead *C. gigas* than *S. glomerata*. The reduced settlement of larvae on high densities of filtering *C. gigas* than *S. glomerata* may be because the strong filtration currents produced by *C. gigas* result in some *S. glomerata* larvae being ingested and trapped or killed by the adult oysters. In the Oosterschelde estuary, Germany, an increase in the suspension feeding biomass following invasion of *C. gigas* reduced the abundance of the mussel *Mytilus edulis* due to consumption of the mussel's larvae (Troost et al. 2009). Our study did not, however, examine the gut contents of *C. gigas* and *S. glomerata* for *S. glomerata* larvae, which would be required to test this hypothesis (see Troost et al. 2008a).

The explanation for the reverse pattern in the field deployments of greater numbers of larvae settling on monospecific patches of C*. gigas* than *S. glomerata* was less clear. Although many species of oyster use waterborne chemical cues to select substrata for settlement (Turner et al. 1994; Zimmer-Faust and Tamburri 1994; Anderson 1996; Tamburri et al. 2008), and transport of cues may be enhanced by currents (Burke 1986), experiments suggested that cues from neither *S. glomerata* nor *C. gigas* influenced settlement of *S. glomerata* larvae (E. M. Wilkie, unpubl. data). Under conditions of flow, oyster patch size, shape, and orientation can each be important factors that influence settlement rates, due to their influence on processes of larval transport (Bell et al. 1995; Tanner 2003; Grabowski et al. 2005). It is possible that the deeper cup-shape of *C. gigas* may intercept currents differently to *S. glomerata*, in such a way that enhances passive deposition of larvae. Alternatively at low densities, and in conditions of low flow, the stronger ciliary currents of *C. gigas* than *S. glomerata* may be more effective at drawing in passing larvae in and promoting larval settlement. Ciliary currents are known to play an important role in determining encounter rates between larvae and adults (Tamburri et al. 2007) and, in the field, more larvae settled on live than dead oysters of each species. Subtle differences in shell microstructure between the two species do not appear to play a role. In experiments utilizing dead oysters, settlement of *S. glomerata* larvae displayed the same density-dependent pattern of increase in settlement with increase in shell area irrespective of the oyster species that was present.

Our observation that more larvae settled on *C. gigas* than *S. glomerata* in field experiment was unexpected given that *S. glomerata* settle gregariously. Indeed, many other marine invertebrates and fishes display greater settlement on conspecifics than on bare or dissimilar habitats (Dobretsov and Wahl 2001; Scardiña et al. 2009), different families of the same order (Booth 1992) and even different genotypes of their species (Bierne et al. 2003). In the field, however, the pattern of greater larval settlement on *C. gigas* than *S. glomerata* disappeared when the non-native oyster was interspersed with *S. glomerata*. This indicates that at the patch scale, a threshold density of *C. gigas* was required for positive effects of the non-native to be seen.

This study considered only artificially manipulated oysters in aquaculture settings. Nevertheless, given that these cultured oysters were derived from wild-caught oysters and our field deployments were in oyster farms adjacent to wild oyster populations, it is likely that the results would also apply to equivalent densities of wild oysters on natural substrates. Recent surveys of wild oyster populations in several east Australian estuaries indicate that densities of *C. gigas* at the estuary- and site-scale remain low. A 2006 survey found that in the Hawkesbury River estuary, the mean density of *C. gigas* on rocky shores was 17 ± 13/m^2^ (Summerhayes et al. 2009). In Port Stephens, the mean density of *C. gigas* in 2008 was 4 ± 1/m^2^ and had not increased since the early 1990s (Bishop et al. 2010). In each estuary, *C. gigas* typically constituted <5% of oysters at a site, and even in the most invaded areas *C. gigas* constituted <20% of total oyster abundance (Summerhayes et al. 2009; Bishop et al. 2010). At these densities, and assuming that our results are applicable to wild oyster populations, negative impacts of *C. gigas* on larval settlement of the magnitude that have been seen in the Wadden Sea and Oosterchelde estuary (Smaal et al. 2005; Troost et al. 2009) are unlikely. Instead, where the two species coexist in well-mixed waters, positive effects of the non-native oyster on *S. glomerata* settlement may be seen. At the patch scale, however, negative impacts of *C. gigas* on *S. glomerata* larval settlement may occur low in the intertidal zone, where *C. gigas* experiences a growth advantage over *S. glomerata* (Krassoi et al. 2008) and locally dominates oyster patches (Bishop et al. 2010). Sampling of mixed oyster beds of *C. gigas* and *S. glomerata* at the patch scale revealed that along a tributary of the Hawkesbury River, NSW the abundance of oyster spat, *Bembicium auratum, Patelloida mimula,* and *Irus crenatus* each decreased with the increasing proportionate contribution of *C. gigas* to total oysters (Wilkie et al. 2012). The observation of reduced larval settlement in wild oyster assemblages dominated by *C. gigas* is consistent with our results from manipulation of cultured oysters. Experiments manipulating oyster density and spatial configuration in natural settings would, however, be required to confirm that the mechanism by which this pattern arises is the same in natural settings.

This study did not investigate the settlement response of *C. gigas* larvae to variation in the density and spatial arrangement of adult substrates. Nevertheless, the inability, like many bivalve larvae, of larval *C. gigas* to detect and escape from inhalant bivalve feeding currents (Troost et al. 2008b) suggests that it may display similar density-dependent differences in settlement between adult substrates as native oyster larvae. *C. gigas* settle on farmed *S. glomerata* in significant numbers, causing over-catch issues for farmers (G. Diemar, pers. comm.). Nevertheless, further experiments would be needed to confirm patterns and drivers of *C. gigas* settlement and whether, if the non-native species is displaying similar settlement patterns to the native oyster, there is competition between new recruits of the two species.

In summary, our study has demonstrated that the small-scale density and distribution of an invader can have a large influence on its impact. Hence, successful management of invaders requires not only knowledge of their broad scale distribution and population size, but also of their small-scale spatial arrangement and how, this in turn influences impacts to native communities. Where the small-scale distribution is more important than the large-scale distribution in determining impact, nonlinear density-dependent effects of non-native species on native biota may occur (e.g., Finnoff et al. 2005; Yokomizo et al. 2009). In order to set realistic and cost-effective goals for management of invaders and their impacts, such density- and scale dependencies need to be understood (Yokomizo et al. 2009). Particularly in instances where relationships between invader density and impact are nonlinear, ignorance of the density–impact curve will lead to goals and management strategies that are mis-matched.

## Supporting Information

**Data S1.** Additional data have been deposited in DRYAD, doi:10.5061/dryad.v7m3q.
