# Supplementary material for: The density and spatial arrangement of the invasive oyster Crassostrea gigas determines its impact on settlement of native oyster larvae
Source: Ecol Evol. 2013 Dec 10;3(15):4851–60. doi: 10.1002/ece3.872 (PMC3892352; doi:10.1002/ece3.872)
Supplement: Supplementary file 1 [file ece30003-4851-SD1.docx]

**Data S1.** Additional data has been deposited in DRYAD, doi:10.5061/dryad.v7m3q
